# Anti-hepatitis B virus (HBV) response of imiquimod based toll like receptor 7 ligand in hbv-positive human hepatocelluar carcinoma cell line

**DOI:** 10.1186/s12879-017-2189-z

**Published:** 2017-01-14

**Authors:** Dipanwita Das, Isha Sengupta, Neelakshi Sarkar, Ananya Pal, Debraj Saha, Manikankana Bandopadhyay, Chandrima Das, Jimmy Narayan, Shivaram Prasad Singh, Sekhar Chakrabarti, Runu Chakravarty

**Affiliations:** 1 ICMR Virus Unit, Kolkata, ID & BG Hospital Campus, ICMR Virus Unit, GB 4, 700010 Kolkata, India; 2 Biophysics & Structural Genomics Division, Saha Institute of Nuclear Physics, Kolkata, India; 3 Department of Gastroenterology, SCB Medical College, Cuttack, India; 4 Kalinga Gastroenterology Foundation, Beam Diagnostics Premises, Cuttack, India; 5 National Institute of Cholera and Enteric Diseases, Kolkata, India

**Keywords:** Hepatitis B virus, TLR7, Innate immune response, Epigenetics, Cell-cycle arrest

## Abstract

**Background:**

Toll like receptors (TLRs) play an important role in innate immunity and various studies suggest that TLRs play a crucial role in pathogenesis of hepatitis B virus (HBV) infection. The present study aims in looking into the status of crucial host and viral gene expression on inciting TLR7.

**Methods:**

The transcription of TLR7 pathway signaling molecules and HBV DNA viral load were quantified by Real Time-PCR after stimulation of TLR7 with its imiquimod based ligand, R837. Cell cycle analysis was performed using flow-cytometry. Expression of TLR7 and chief cell cycle regulator governing G1/S transition, p53 was also seen in liver biopsysss samples of CHB patients. HBV induced alteration in histone modifications in HepG2 cells and its restoration on TLR7 activation was determined using western blot.

**Results:**

The TLR7 expression remains downregulated in HepG2.2.15 cells and in liver biopsy samples from CHB patients. Interestingly HBV DNA viral load showed an inverse relationship with the TLR7 expression in the biopsy samples. We also evaluated the anti-viral activity of R837, an agonist of TLR7. It was observed that there was a suppression of HBV replication and viral protein production upon TLR7 stimulation. R837 triggers the anti-viral action probably through the Jun N-terminal Kinase (JNK) pathway. We also observed a downregulation of histone H3K9Me3 repression mark upon R837 treatment in HBV replicating HepG2.2.15 cells, mimicking that of un-infected HepG2 cells. Additionally, the G1/S cell cycle arrest introduced by HBV in HepG2.2.15 cells was released upon ligand treatment.

**Conclusion:**

The study thus holds a close insight into the changes in hepatocyte micro-environment on TLR7 stimulation in HBV infection.

**Electronic supplementary material:**

The online version of this article (doi:10.1186/s12879-017-2189-z) contains supplementary material, which is available to authorized users.

## Background

Hepatitis B virus (HBV) infection is the leading cause of liver cirrhosis and hepatocellular carcinoma (HCC); however the outcome of the infection varies widely among infected individuals [1]. Although various therapies including alpha interferon and nucleoside/-tide analogues are in use for treating HBV infection, a constant effort is being made to develop more potential and cost effective drugs.

Innate immunity, the first line of defense, plays a vital role in limiting the spread of pathogen after the initial infection and triggers an effective adaptive immune response. It has been seen that viral particles and its components are sensed by pattern recognition receptors (PRR), which include the RIG-I-like receptors (RLRs), nucleotide oligomerization domain (NOD)-like receptors (NLRs) and the toll-like receptors (TLRs). They subsequently activate an effective signaling pathway, which is responsible for the production of antiviral cytokines. Though a clear role of adaptive immune response has been seen in HBV clearance; the role of innate immunity in HBV infection still remains enigmatic [2]. Earlier, it was assumed that HBV was unable to induce an innate immune response by acting as a ‘stealth virus’, which skillfully evades the first line of defense and strategically block important candidates in its signaling pathway [3]. Therefore, HBV remains undetected by the host immune surveillance and infects the hepatocytes until the adaptive immunity is triggered weeks later. However, on the contrary, HepaRG cells as well as SCID mice harboring humanized liver, shows an up-regulation of IFN (Interferon) response upon HBV infection [4, 5]. Different TLR agonists have been clinically assessed for treatment of chronic viral infections like HBV, Hepatitis C virus (HCV) and Human Immunodeficiency Virus (HIV) [6]. Previous studies have shown that TLR3, TLR4, TLR5, TLR7, and TLR9 ligands/agonists when administered intravenously in HBV transgenic mice, inhibits HBV replication [7]. In addition, a recent study showed that activation of TLR2 is instrumental in suppression of HBV replication in hepatoma cell lines and woodchuck models [8].

Single stranded viral RNAs and synthetic compounds like imidazoquinoline, loxoribine serve as agonists for TLR7, which mainly operates through the Myeloid Differentiation primary-response protein88 (MyD88) dependent pathway. The subsequent messengers in the signaling pathway activate different transcription factors including Nuclear Factor kappa-light-chain-enhancer of activated B cells (NF-КB), Jun N-terminal Kinase (JNK) etc. that turns on the expression of downstream targets and inflammatory cytokine secreting genes. In the present study we have tried to look into the antiviral role of TLR7 in hepatocyte microenvironment during HBV infection. TLR7 exhibits viral clearance by modulating several key host factors. Cell cycle analysis was carried out to check the fate of HBV induced G1/S arrest on TLR7 activation. We also investigated the epigenetic alteration as a sequel to HBV infection and monitored a partial reversal upon TLR7 agonist treatment implicating an alteration of gene expression.

## Method

### Study subjects

A total of 19 liver biopsy samples were collected from patients at Kalinga Gastroenterology Foundation (Cuttack, Orissa, India) of which, 12 individuals had chronic HBV infection. 7 biopsy samples were taken from patients with steatosis but had no history of HBV, HCV or HIV infections and they were taken as disease controls. Signed informed consent was obtained from patients and the study was approved by the institutional ethical committee. The diagnosis of patients with CHB was conformed according to the AASLD guidelines 2009.

### Cell culture

The maintenance and plating of hepatoblastoma cell lines HepG2 and HepG2.2.15 was done as described previously [9].

### Lamivudine treatment

Lamivir (Lamivudine tablets-150 mg) were provided by Cipla. The tablets were suspended in sterile water and then filtered using 0.2-μm filters. Cells were treated with 10, 20, 50 and 100 μM (final concentration) of Lamivudine every day for 72 h as shown previously [10], after which cells were harvested for further analysis.

### Analysis of HBV viral properties

The synthetic ligand used for TLR7 was Imiquimod-R837 provided by Invivogen. Cell culture supernatant was collected from HepG2.2.15 cells after treatment with 4, 6 and 8 μg/ml of R837 for 72 h. Total DNA was extracted using Qiagen Blood mini-kit (Hilden, Germany). HBV viral load in culture supernatant was quantified by real-time TaqMan PCR assay using NIBSC standards as described earlier [11]. HBeAg and HBsAg levels were analyzed by using commercial ELISA kits (Diasorin, S.P.A., Saluggia, Italy).

### Chemical inhibitors

For pathway screening, HepG2.2.15 cells were stimulated with 8 μg/ml of R837 (Invivogen, San Diego, CA, USA) singly or in conjunction with various inhibitors. 10 μM NF-КB pathway inhibitor PDTC, 25 μg/mL of SP600125 (MAPK/JNK pathway inhibitor), 2μMof LY294002 (inhibitor of PI3K) and 10 μM SB203580 (MAPK/p38 pathway inhibitor) were used. They were purchased from Sigma–Aldrich (St. Louis, MO, USA). These inhibitors have specific targets and block the exact pathways that they are chosen for [12].

### RT-PCR analysis

For the RNA expression from HepG2 and HepG2.2.15 cells, 1x10^6^ cells were plated in 6-well plate for the different experiments. For the mRNA expression of TLR7, different cell cycle regulators and the different TLR7 signaling molecules from HepG2/HepG2.2.15 cells, total RNA was treated with DNase and was reverse transcribed with random hexamers using Revert Aid first-strand cDNA synthesis kit (MBI Fermentas). Real time PCR was performed in ABI 7200 SDS (Applied Biosystems, Foster City, CA, USA) using Power SYBR Green (Applied Biosystems). The target mRNA was relatively quantified and was normalized to the internal control (GAPDH). The PCR cycle number (C_T_) at which the exponential growth in the fluorescence from the dye (SYBR Green) passes a certain threshold was used to calculate the relative gene expression. 2^-ΔΔCT^ was calculated to represent the relative quantification of the gene, where Δ*C*_T_ (*C*_T-target gene_ – *C*_T-GAPDH_). ΔΔCT = Δ*C*_T (Experiment) -_ Δ*C*_T(Control)._ List of primers used are listed in Table 1.

### Western blot analysis

For western blotting, cells were harvested and lysed with Laemelli buffer containing 120 mMTris-HCl (pH-6.8), 20% Glycerol and 4% SDS. Almost equal amounts of protein were then run in a SDS PAGE and transferred on Nitrocellulose membrane (Millipore). Following incubation with primary antibody (overnight at 4 °C) and HRP conjugated secondary antibody (3 h at room temperature), the blots were developed using chemiluminescent substrate (Millipore). Densitometry measurements of bands were used for quantification of each marker by integrating each peak in Image J software. List of primary antibodies are listed in Table 2.

**Table 1:** List of primer sequences used for RT-PCR analysis

Gene	FORWARD PRIMER	REVERSE PRIMER
IRAK 1	5'-ACCGCAGATTATCATCAACC-3'	5'-AGACTTACAGCCATACTTCACT-3'
IRAK 4	5'-GCTGTATGTAGGGTGGAAAC-3’	5'-TGCTGACAACTGGAAGGTAG-3'
TRAF6	5'-GCCCAGGCTGTTCATAGTTT-3'	5'-CAAGGGAGGTGGCTGTCATA-3'
p53	5’-CCCAAGCAATGGATGATTTGA-3’	5’-GGCATTCTGGGAGCTTCATCT-3’
CYCLIN D1	5’-AGCTCCTGTGCTGCGAAGTGGAAAC-3’	5’-AGTGTTCAATGAAATCGTGCGGGGT-3’
CYCLIN E	5’-CAGCACTTTCT TG AGCAACACCCTC-3’	5’-TCTCTAT GTCGCACCACTGATACCC-3’
CYCLIN B1	5’-AAGAGCTTTAAACTTTGGTCTGGG-3’	5’-CTTTGTAAGTCCTTGATTTACCATG-3’
CYCLIN A	5’GCATGTCACCGTTCCTCCTT-3’	5’CAGGGCATCTTCACGCTCTAT-3’
JNK	5'- GTACTTGTATGAAACCACCTTTCT -3'	5'- AGCATCTCTTTCTGAATCTATGAAG -3'
PI3KCA	5'- AAGGGTGCTAAAGAGGAACAC -3’	5'-CATGAGGTACTGGCCAAAGAT -3'
PI3KCB	5'- CTCCAAATGTTGCGCTTGATG -3'	5'- ACAACTTCAATGAGGCCAGAG -3'
PI3KCG	5’- CACCGAGACAGGAAACCTATTT -3’	5’- TAGCACAAATGGCACTCTCTC -3’
NF-КB	5’-CGCATCCAGACCAACAACA-3’	5’- TGCCAGAGTTTCGGTTCAC-3’
p38	5’- TCTGCTTACCCTTCACCTTTG -3’	5’- CACATCCTCACTCTGCTAGAAAT -3’
c-jun	5’-CAAAGTTTGGATTGCATCAATG-3’	5’- TAACATTATAAATGGTCACAGCACATG-3’
c-myc	5’-CTTCTCTCCGTCCTCGGATTCT-3’	5’-GAAGGTGATCCAGACTCTGACCTT-3’
Sap-1	5’-GCTTTTGCCACCACACCACCCATTTCG-3’	5’-GCCCAGACAGAGTGAATGGCCCATGAC-3’
Elk-1	5’-ACCTGAAATCGGAAGAGCTTAAT-3’	5’-AACTTCCAACTCTTCCTTGGG-3’
TNF-α	5’-ATGGGCTACAGGCTTGTCACT-3’	5’-CTCTTGGCAGCCTTCCTGATT-3’
IFN-β	5’-GTCTCCTCCAAATTGCTCTC-3’	5’-ACAGGAGCTTCTGACACTGA-3’
IFN-α	5’-TGGCTGTGAAGAAATACTTCCG-3’	5’-TGTTTTCATGTTGGACCAGATG-3’
IL-6	5’-ATGTAGCCGCCCCACACAGA-3’	5’-CATCCATCTTTTTCAGCCAT-3’
IL-1β	5’-ACAGATGAAGTGCTCCTTCCA-3’	5’-GTCGGAGATTCGTAGCTGGAT-3’
GAPDH	5'-AAGGCTGTGGGCAAGG-3'	5'-TGGAGGAGTGGGTGTCG-3'

**Table 2:** List of Antibodies used

ANTIBODY	CAT. NO.	COMPANY
Anti-GAPDH	ab9485	ABCAM
Anti-H3	ab10799	ABCAM
Anti-H3K36Me3	ab9050	ABCAM
Anti-H3K4Me3	39159	ACTIVE MOTIF
Anti-H3K9Me3	39161	ACTIVE MOTIF
Anti-H3K27Me3	39155	ACTIVE MOTIF
Anti-H3K18Ac	39587	ACTIVE MOTIF
Anti-H3K9Ac		MILLIPORE,07-352
Anti-NFКBp65	14-6731-81	e-BIOSCIENCE
Anti-p53	SC-126	SANTA CRUZ BIOTECHNOLOGY
Anti-Mouse IgG-HRP	W402B	PROMEGA
Anti-Rabbit IgG-HRP	A1949	SIGMA
Anti-Rabbit Alexa Fluor	A11034	INVITROGEN

### Confocal imaging to detect NF-КB nuclear translocation

HepG2.2.15 cells were grown on coverslips and treated with R837 as stated earlier. The cells were washed thrice with Phosphate buffered saline (PBS) and then fixed with 4% Paraformaldehyde (Sigma) in PBS for 10 min at room temperature (RT). Cells were washed thrice with PBS and permeabilized with 1% Triton X-100 in PBS for 10 min at RT, washed with PBS thrice followed by blocking with 3% BSA in PBS for 1 h at RT. Cells were incubated with Anti-NFКB (eBioscience) for 1 h at RT, washed thrice with PBST (PBS + 0.05% Tween20) and then incubated with secondary antibody (Alexa-Fluor anti-rabbit 488, Invitrogen) for 1 h in dark at RT, followed by three washes with PBST. Coverslips were mounted with mounting media containing DAPI (Sigma Aldrich). Fluorescence for Alexa and DAPI was visualized with Nikon Ti-E confocal microscope with A1RMP scanner head equipped with Nikon imaging software (NIS).

### Cell cycle analysis

For cell cycle analysis, the cells were harvested and thoroughly re-suspended in Phosphate Buffered Saline (PBS). The cells were then fixed by adding double volume of chilled 70% ethanol (Merck) drop wise, with continuous vortexing. After incubating the mixture overnight at −20 °C, it was spinned and the cells were resuspended in 500 μl of PBS. The cells were then incubated with RNaseA (0.2 mg/ml) for 30 min followed by Propidium Iodide (50 μg/ml) (Sigma) at 37 °C for 1 h. Flow cytometric data acquisition was performed on BD FACS Calibur platform.

### MTT assay

Cell proliferation was measured by MTT assay (Cell Titer 96® AQueous One Solution Cell Proliferation Assay, Promega, USA) as described previously [9]. The cells were uniformly seeded in each well of 96-well plates and grown in RPMI 1640 supplemented with 10% FBS. After 24 h, the media was removed and replaced with fresh media and treated with R837. The plates were incubated at 37 °C in a humidified atmosphere of 5% CO_2_ for 72 h. At indicated time-points, relative cell numbers were determined by incubating cells with MTT3-(4, 5-dimethylthiazol-2-yl)-2, 5-diphenyltetrazolium bromide. The absorbance at 490 nm is directly proportional to the number of viable cells. All experiments were performed in triplicates.

## Results

### Reduced TLR7 expression during HBV infection

**Fig. 1:**
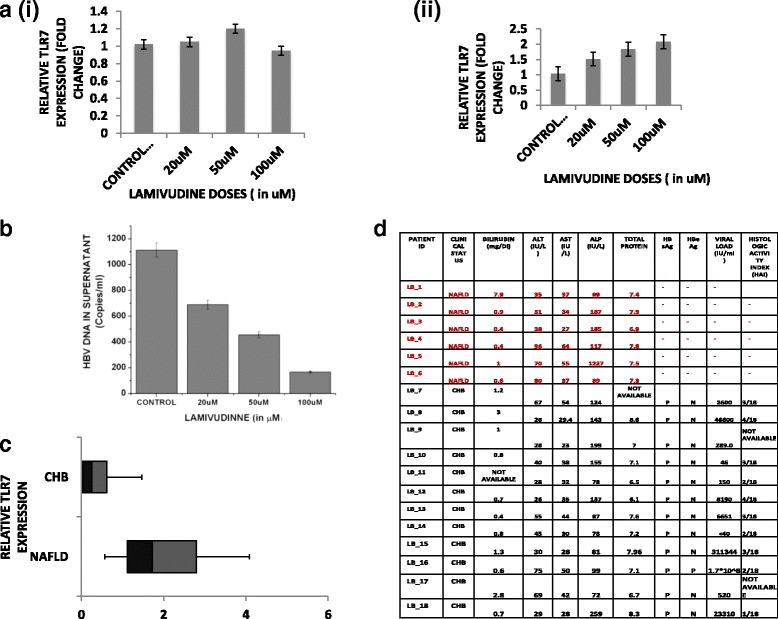
TLR7 expression remains compromised in HepG2.2.15 cell lines. The expressions are significantly re-established on viral elimination. TLR7 expression of HBV infected subjects is downregulated compared to disease control steatosis patients and is also inversely related to the HBV DNA load in liver biopsy samples. **a** TLR7 mRNA expression of HepG2.2.15 cells is regained on treatment with Lamivudine, a potent nucleoside analog reverse transcriptase inhibitor, at different doses (20, 50 and 100μM). **a** TLR7 mRNA expression of HepG2 cells taken as control after treatment with Lamivudine shows no change. **b** TLR7 expression in HepG2.2.15 cells on treatment with different doses of Lamivudine. **b** HBV DNA viral load is suppressed on treatment of Lamivudine in a dose dependent manner. **c** The mean TLR7 expression of the control subjects was arbitrarily set as 1. The fold change of TLR7 expression in each of the patients was then evaluated. Increased TLR7 expression of liver biopsy specimen of control subjects with steatosis (LB_1-6) compared to HBV infected patients (LB7_18) along with clinical and virological parameters of the patients. Box and Whisker Plot was used to score the TLR7 expression. **d** Viral and clinical parameters of the patients

Our previous studies have showed that TLR7 mRNA expression is highly compromised in HBV replicating HepG2.2.15 cells when compared to the HepG2 cells [9]. It is believed that HBV represses the innate immune response during a natural infection. Thus, it was expected that TLR7 expression would be re-established on viral elimination. We therefore, wanted to check the TLR7 expression of HepG2.2.15 cells on administration of a common nucleoside analogue (Lamivudine). It was seen that TLR7 expression showed a gradual increase on treatment of HepG2.2.15 cells with Lamivudine in a dose dependent manner (20 μM, 50 μM and 100 μM). The doses of lamivudine were used previously where HBV mutants were subjected to drug susceptibility assay in vitro [10]. HepG2 cells taken as control, did not show changes in TLR7 expression on Lamivudine treatment (Fig. 1a i and ii). We also showed a significant suppression of HBV DNA viral load on Lamivudine treatment (Fig. 1b). This broadly indicated that HBV viral load was inversely proportional to the TLR7 expression. Small-scale preliminary study also showed that TLR7 expression was downregulated in liver biopsy specimens from chronic HBV patients (LB_7-18) (*n* = 12) compared to control steatosis individuals (LB_1-6) (*n =* 6) (Fig. 1c). The viral and clinical parameters of the patients are also listed (Fig. 1d). Interestingly, while analyzing the TLR7 expression in liver biopsy specimens, it was seen that the HBV viral loads in the biopsies showed an inverse relationship with the TLR7 expression status. Liver biopsy samples LB_8, LB_13, LB_15, LB_16, LB_18 exhibiting high viral loads had lower TLR7 levels. We thus wanted to investigate whether inciting TLR7 with its specific ligand could help in reduction of viral components.

### Suppression of HBV replication upon TLR7 activation in HepG2.2.15 cells

**Fig. 2:**
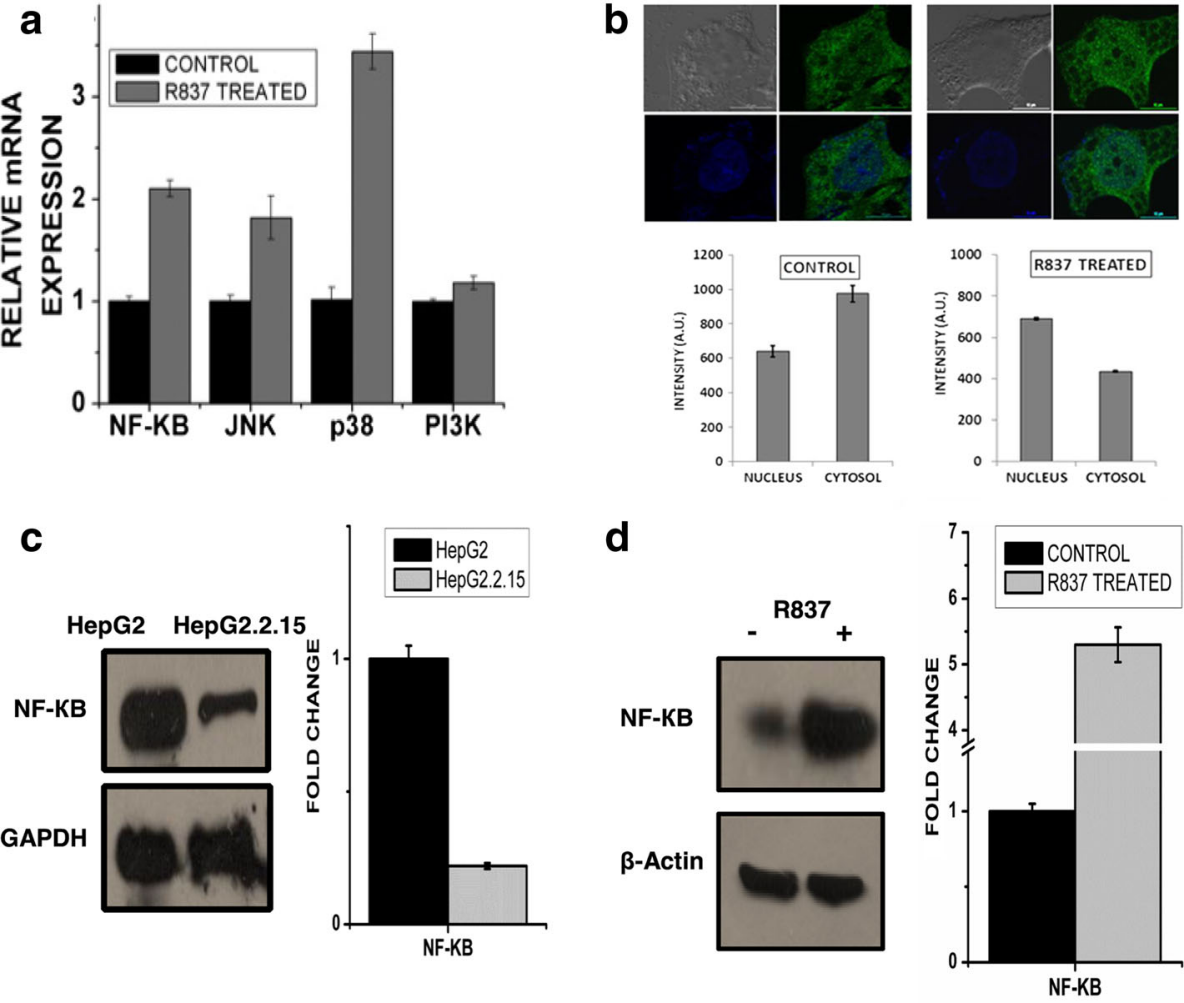
TLR7 pathway is activated on stimulation with its specific ligand imiquimod, R837. Chief downstream regulators, especially NF-ĸB is activated and transclocated to the nucleus on TLR7 activation. **a** Host immune response on TLR7 activation, using TLR7 ligand. HepG2.2.15 cells were treated with TLR7 agonist, R837 (8μg/ml) for 3 days. mRNA expression of different downstream regulators (NF- КB, JNK, p38 and PI3K) involved in the TLR7 pathway. **b** Confocal imaging showing nuclear uptake of Nuclear Factor-КB p65 subunit (NF- КB) from cytoplasm in cells where HepG2.2.15 cells are taken as control and HepG2.2.15 cells stimulated with TLR7 ligand, R837 (8μg/ml) for 72 h. **c** Nuclear Factor-КB p65 subunit (NF-КB) protein expression of HepG2 and HepG2.2.15 cells. **d** Nuclear Factor-КB p65 subunit (NF- КB) protein expression of HepG2.2.15 cells taken as control and treated with R837 (8μg/ml) for 72 h

**Fig. 3:**
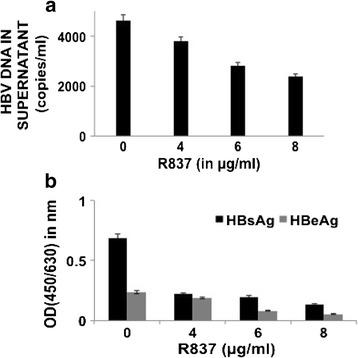
HBV titre was evaluated in culture supernatants of HepG2.2.15 cells after treatment with 4, 6 and 8μg/ml of TLR7 agonist (R837) for 72 h. **a** HBV DNA was isolated from the culture media of R837 treated cells and the load was assessed by absolute real-time PCR using WHO standards. **b** HBsAg and HBeAg were detected from the culture supernatant of treated cells by ELISA

R837 activates a MyD88-dependent TLR7 pathway and leads to the induction of different transcription factors, including NF-КB. To confirm the stimulation of TLR7 with its ligand imidazoquinoline (R837), the expression of chief down-stream molecules belonging to the TLR7 pathway were checked post treatment. Stimulation led to increased expression of the different pathway regulators (NF-КB, p38, JNK). However, PI3K expression did not show a significant change on TLR7 stimulation (Fig. 2a). Chief antiviral cytokines (IFN-α, IFN-β, TNF-α and IL-6) which are associated with HBV clearance were also upregulated on TLR7 stimulation (Fig. S1A). Stimulation of TLR7, promoted proteolytic degradation of the inhibitor I-КB and allowed nuclear translocation of Nuclear Factor-КB p65 subunit from the cytoplasm of the cells (Fig. 2b). Interestingly, the protein expression of NF-КB is greatly compromised in HepG2.2.15 cells compared to HepG2 cells (Fig. 2c), the levels of which are substantially restored upon TLR7 activation (Fig. 2d). In an attempt to address the effect of TLR7 activation in HBV replication, HepG2.2.15 cells were treated with 4, 6 and 8 μg/ml of R837 for 72 h and there was a significant decrease in HBV DNA viral load (Fig. 3a) and viral protein (HBsAg and HBeAg) concentration (Fig. 3b) in a dose dependent manner in the culture supernatant upon treatment with ligand.

### TLR7 agonist suppresses HBV replication through JNK pathway

**Fig. 4:**
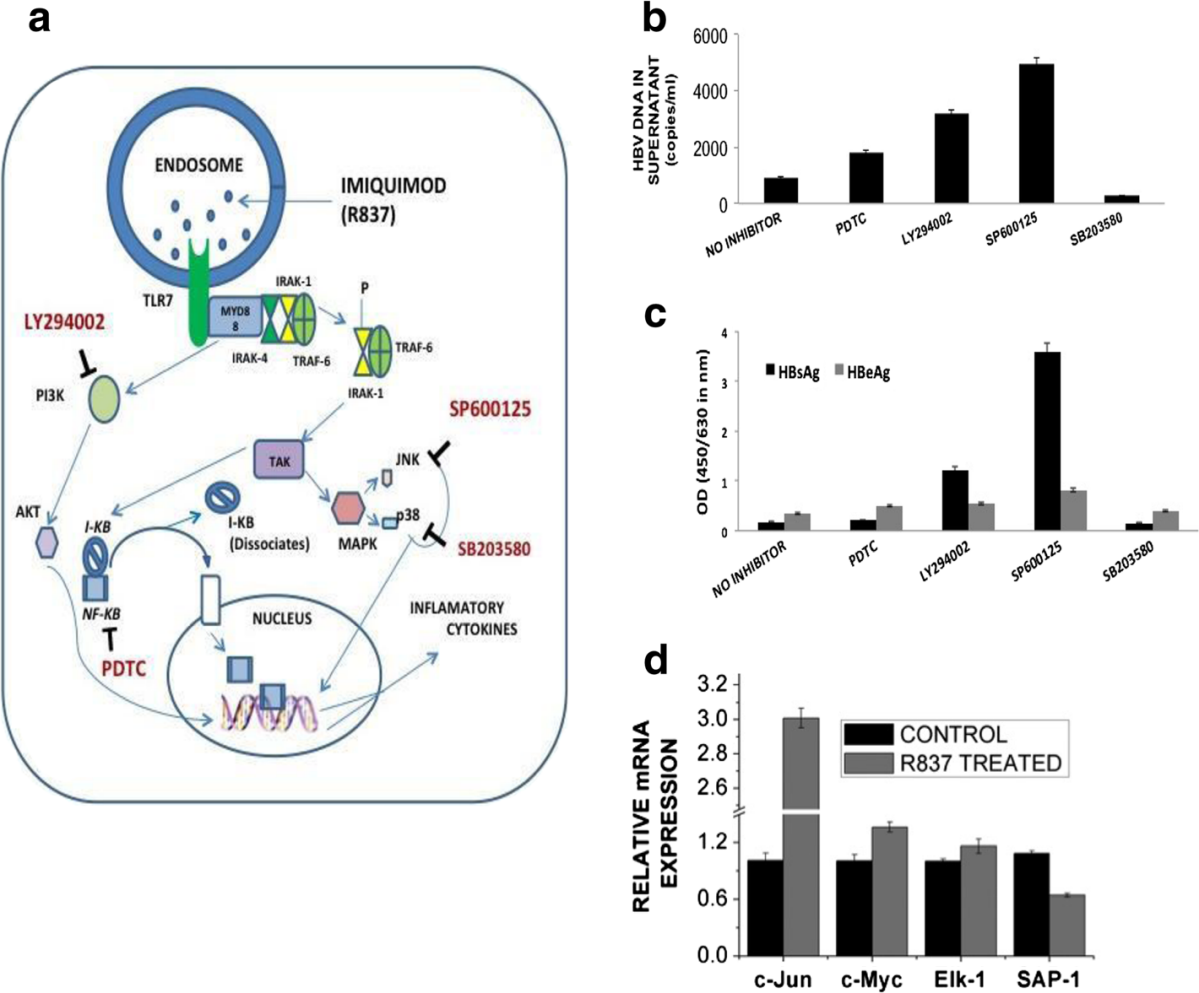
TLR7 mediated cellular signaling pathway in suppression of HBV replication. HepG2.2.15 cells were pretreated with or without different pathway inhibitors (NF-КB pathway inhibitor PDTC, MAPK/JNK pathway inhibitor SP600125, PI3K-AKT inhibitor LY294002 and MAPK/p38 pathway inhibitor SB203580). After 1 h incubation, R837 was treated for 6 h. **a** Schematic representation of TLR7 signaling pathway and important inhibitors of relevant signaling molecules. **b** and **c**. HBV DNA, HBsAg AND HBeAg from culture supernatants on stimulation with R837 alone or pre-treated with different pathway inhibitors detected by real time RT- PCR and ELISA. **d**. Relative mRNA expression of JNK–dependent/ independent responsive genes (c-Jun, c-Myc, Elk-1 and SAP-1)

A look at the signaling cascade involving TLR7 is depicted in Fig. 4a. In order to identify the pathway through which TLR7 executes its anti-viral effect, we targeted and inhibited some key molecules essential in each of these pathways (JNK, p38, PI3K and NF-КB) in presence of R837. HBV DNA load was maximal when the JNK pathway was blocked with SP600125 even when TLR7 was stimulated as compared to the other blockers PDTC, LY294002 and SB203580 which blocked NF-КB, PI3K and p38 pathway respectively (Fig. 4b). These blockers have specific targets and retards definite signaling pathways. Our findings thus suggest that JNK pathway blocker SP600125 impedes the antiviral action of R837. Increased viral protein expression (HBsAg and HBeAg) on using SP600125 substantiates the observation, that TLR7 agonist shows its antiviral action through the JNK pathway (Fig 4C). The host cytokine profile (chiefly associated with HBV clearance) was monitored in presence of protein blockers in combination with R837 in order to identify the cytokines responsible for viral suppression. The expression of antiviral cytokines including IFN-β, TNF-α, IL-1β, IL-6 and IL-10 was substantially repressed on blocking JNK pathway with its inhibitor. However, the expression of the cytokines IFN-α and IFN-γ showed no significant downregulation (Additional file 1: Fig. S1b). Thus, the cytokines IFN-β, TNF-α, IL-1β, IL-6 and IL-10 probably play a crucial role in HBV suppression on TLR7 activation. R837 modulates the key components of signal transduction pathway of host cells and implicate a broad-spectrum function of this agonist in its ability to modulate multiple cellular functions.

### TLR7 stimulation exerts an alteration in host epigenetic signatures in HepG2.2.15 cells

We have monitored that TLR7 activation affects the transcription of multiple downstream targets including NF-КB, p38 and JNK. All these proteins are important hubs of signal transduction pathway and can execute pleiotropic effects. We have observed antiviral response of TLR7 is majorly triggered through the JNK pathway. We thus wanted to monitor the alteration of expression of several JNK downstream targets following TLR7 activation. Interestingly, JNK responsive gene c-Jun showed prominent increase in transcription unlike SAP-1 whose expression is in a JNK independent manner. Other JNK responsive genes like c-Myc and Elk-1 [13] showed marginal changes (Fig. 4d), indicating that the antiviral response although majorly steered through JNK pathway, is mostly gene specific.

**Fig. 5:**
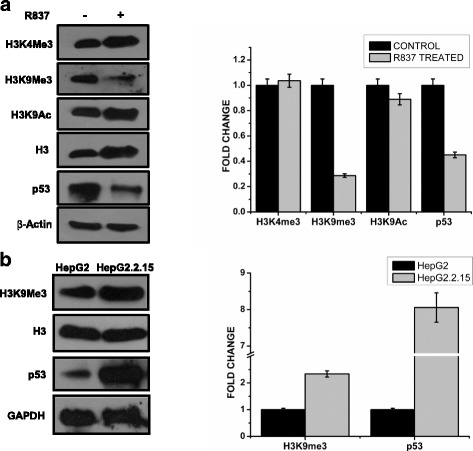
Status of epigenetic markers and transcription factors on TLR7 activation. HepG2.2.15 cells were treated with R837 for 72 h and then the cells were subjected to Western Blot for protein expression studies. **a** Protein expression of different histone modifications (H3K4Me3, H3K9Me3, H3K36Me3 and H3K9Ac) and p53 before and after TLR7 activation. **b** The relative expression of the proteins (H3K9Me3 and p53) in HepG2 and HepG2.2.15 cells

Since TLR7 activation upregulates the expression of different downstream regulators, we wanted to understand its role, affecting the transcription of chromatin template. In order to assess that, the global alteration of selective histone modification including H3K4Me3, H3K9Me3 and H3K9Ac, was quantified in HepG2.2.15 cells following R837 treatment (Fig. 5a). While the activation signatures (H3K4Me3, H3K36Me3 and H3K9Ac) did not show a significant change, alteration in H3K9Me3 repression mark was noteworthy which indicates a de-repression scenario where active transcription was re-established. A comparison of these epigenetic signatures between HepG2 and HepG2.2.15 cells was further carried out and H3K9Me3 level, unlike other marks, showed an increased expression in the latter cell line (Fig. 5b). Thus, upon HBV infection host transcription would preferably halt which is evident from the epigenetic change. This repression is again rescued upon TLR7 activation implicating that R837 has an ability to affect the cellular microenvironment thereby re-establishing transcription activation status. Thus the HBV induced alteration of host epigenetic status implicates a novel role of this ligand in regulating cellular transcription profile of host cell.

### TLR7 activation results in the release of G1/S cell cycle arrest in HepG2.2.15 cells

**Fig. 6:**
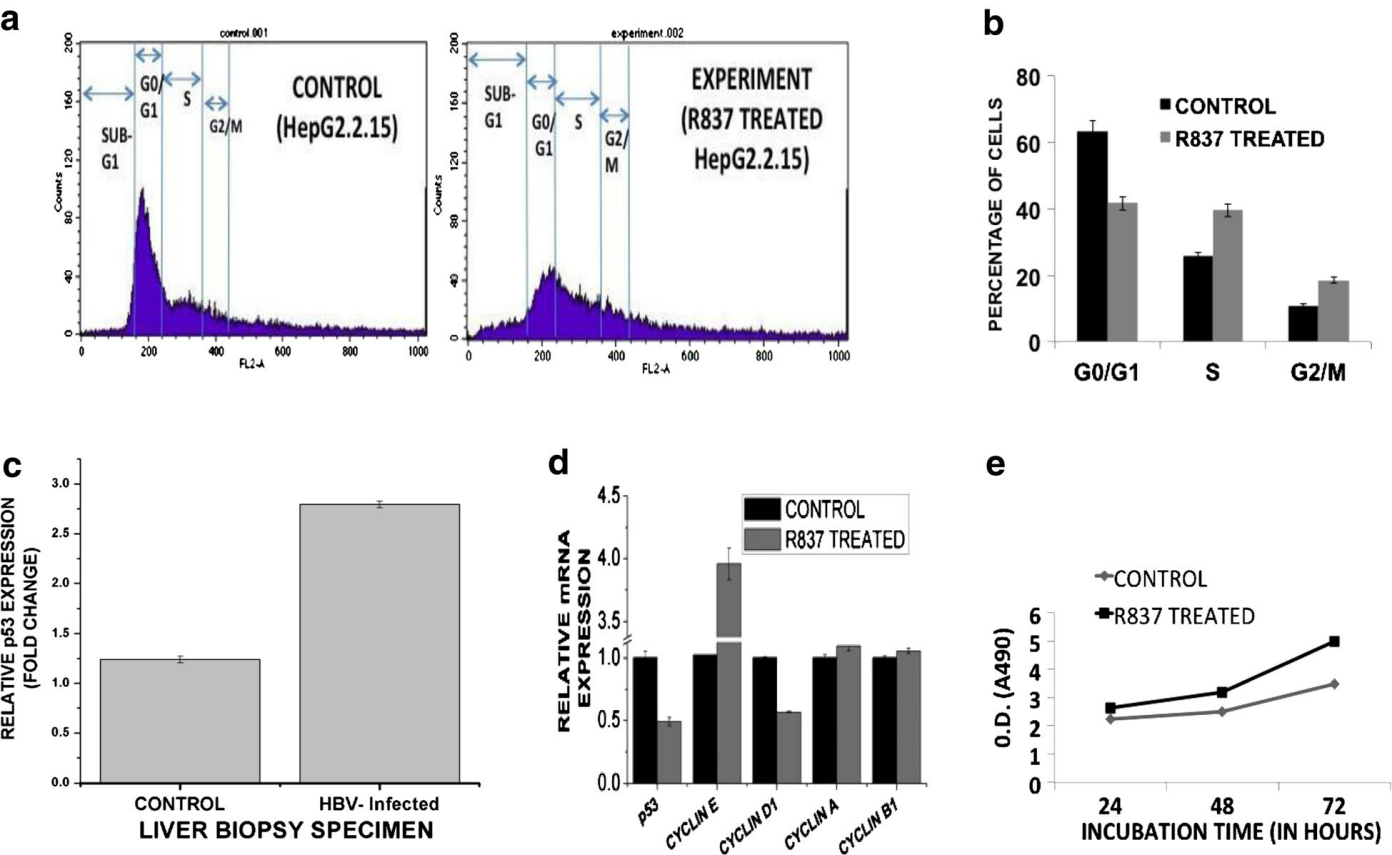
Flow cytometric analysis and alteration of key cell cycle regulators upon TLR7 activation. HepG2.2.15 cells were treated with R837 (8μg/ml) for 72 h and then subjected to flow cytometric analysis to study the status of G1/S arrest. **a** Cell cycle analysis by FACS. **b** Percentage of cells in different phases of cell cycle before and after TLR7 activation. **c** p53 expression in liver biopsy samples of chronic HBV patients compared to control steatosis patients. **d** Relative mRNA expression of different cell cycle regulators (p53, Cyclin E, Cyclin D1, Cyclin B1 and Cyclin A) on R837 treatment. **e** Detection of invitro cell proliferation on TLR7 activation using MTT assay. HepG2.2.15 cells were treated with R837 for 72 h and then incubated with MTT at the indicated time points

The alteration of epigenetic signature in HepG2.2.15 cells as a sequel to R837 treatment implicates a broad-spectrum effect in host cell milieu. We further investigated whether TLR7 activation might influence the host cell cycle stages. It has been shown previously that HBV transformed cell line HepG2.2.15 undergoes a G1/S arrest. This arrest is induced by the virus and results in slow proliferation of the cells [14]. Since TLR7 activation reduces the HBV DNA viral load, we wanted to find out, whether this would affect the status of G1/S arrest within the cells. It was observed that reduction of HBV infection induced a release in the G1/S arrest (Fig. 6a). The percentage of cells in the different phases of cell cycle was determined in HepG2.2.15 cells before and after the treatment with R837 (Fig. 6b). The proportion of cells in G1 phase was maximal compared to S and G2/M phases in HepG2.2.15 cells. A distinct G1/S arrest in the cell cycle of the HepG2.2.15 cells was observed. To assess the antiviral action of R837, we checked the proportion of cells in different phases of the cell cycle on TLR7 activation. The quantity of cells in the G1 phase showed significant reduction, when the cells were treated with R837. The cells apparently entered the S phase after a G1 escape. Thus, a distinct release in the cell cycle arrest in HepG2.2.15 cells was observed after treatment. To investigate the mechanism of G1 phase arrest induced by HBV and subsequent release in arrest after TLR7 activation, the expression of cell cycle regulators was studied. It was expected that the dynamics of transition of different phases of cell cycle would corroborate with expression of cell cycle genes. p53 is the key regulator in the transition from G1 to S phase and is upregulated in virus infected cells [15]. In this connection it was observed that mRNA expression of p53 remains upregulated in liver biopsy samples of chronic HBV patients (*n =* 8) as compared to steatosis patients, serving as disease control (*n =* 7) (Fig. 6c). Further, treated cells showed a reduced mRNA and protein expression of p53 compared to the untreated HepG2.2.15 cells (Fig. 6d, 5B). Cyclin D1 is also required for G1/S cell cycle transition and remains upregulated in HepG2.2.15 cells compared to HepG2 cells (16). TLR7 activation led to a downregulated expression of Cyclin D1 transcript indicating the role of TLR7 in viral reduction. Cyclin E, another crucial regulator of G1/S transition, remains downregulated in infected cells [16]. It was seen that mRNA expression of Cyclin E was significantly upregulated in treated cells (Fig. 6d) implicating a p53- independent cell cycle regulatory pathway upon TLR7 activation. Since the proportion of cells in G2/M transition was not affected after treatment, it was expected that the regulators too would not show significant modulation. Our findings showed, that the cell cycle regulators CyclinB1 and A, which play crucial role in G2/M and late S into late G2 phase transition respectively, had no significant changes in treated and untreated HepG2.2.15 cells (Fig. 6d). Further, MTT assay showed that treatment of HepG2.2.15 cells with R837 at different time points had no significant cytotoxic affects and on the contrary promoted cell growth (Fig. 6e). MTT assay clearly showed that there was no significant cell death due to R837 treatment.

## Discussion

TLRs have a pivotal role in the regulation of innate immune responses and are intricately associated with the host defense mechanism. TLR activation leads to the production of endogenous interferons and antiviral cytokines, which establishes a crosstalk between innate and adaptive immune responses. This triggers the immune effector cells, which plays a vital role in the antiviral immunity. Studies show that modulation of TLR signaling pathways can be effective in controlling infectious diseases, immune-related disorders and cancers [17]. It has been seen previously that the innate immune response remains suppressed on HBV infection [18]. In the current study, we have shown that TLR7 expression is reduced in liver biopsy samples of Chronic Hepatitis B patients compared to steatosis individuals serving as disease controls. Patients with steatosis have been earlier used as disease control for HBV-related innate immune studies [19]. We have also showed that the compromised TLR7 expression in HepG2.2.15 cells was significantly re-established when HBV replication was suppressed by use of a common nucleoside analogue (Lamivudine), thus further proving that HBV suppresses TLR7. Lamivudine is a currently approved licensed anti-HBV drug which results in decrease in the circulating HBV DNA levels [20]. Previous studies have showed that Lamivudine significantly inhibited levels of HBV DNA in HepG2.2.15 cell line [21]. This observation was further confirmed, since there was no significant change in TLR7 expression in HepG2 cells taken as control.

Our previous studies have shown that both TLR7 and miR-155 are down-regulated and positively correlate with each other in HBV infection and ectopic expression of miR-155 can reduce HBV viral load through targeted suppression of C/EBP-β [9]. Thus, possibly, stimulation of TLR7 might as well exhibit an anti-viral effect. Therefore, we aimed to explore the effect of TLR7 stimulation on HBV replication in hepatocyte micro-environment. Our findings show that TLR7 activation leads to suppression of HBV replication and viral protein (HBsAg and HBeAg) production, which is in concordance to a previous study that was conducted in Chimpanzees [22]. This study takes a step forward in identifying the different host factors and the alterations in gene expression on TLR7 activation in virus infected hepatocytes. An earlier report showed that MAPK/ERK and PI3K/AKT pathways were responsible for the suppression of HBV replication through TLR2 stimulation [8], however no such studies were conducted with TLR7. In the current study, we found that the antiviral action of TLR7 takes place through the JNK pathway. JNKs are subgroups of MAP kinases and are activated in response to cytokines, TLRs and antigen receptors like T and B cells [23]. Activated JNKs phosphorylate c-Jun, JunD, ATF and other transcription factors, which is involved in the formation and activation of AP-1 complex. C-jun is a downstream molecule phosphorylated by the JNKs and is a well-characterized oncogene, found in the liver [24]. Results showed that there was an increased expression of c-jun on TLR7 stimulation, indicating the activation of JNK pathway and its possible role in the anti-viral response. Further, it was seen that blocking JNK pathway with SP600125, curbed the expression of different cytokines including IFN-β, TNF-α, IL-1β, IL-6 and IL-10. Chief anti-inflammatory cytokine IL-10 and pro-inflammatory cytokines IL-6 and TNF-α probably play a balanced role in viral elimination. The blockers that have been used for pathway screening have specific roles and act specifically on different substrates. Previously, several cytokines have been shown to have a HBV suppressive effect in infected transgenic mice and cell line models. Several cytokines viz. IL-12, IL-18 and IFNs have been shown to have a suppressive effect in HBV-infected transgenic mice [25–27]. Another study had shown that IFN-γ synergistically acts with TNF-α and inhibits expression of HBV RNA levels in immortalized hepatocyte cell line (HBV-MET) [28]. IL-4 has also been shown to be an effective cytokine in viral protein suppression in Hep3B cell line [29]. Although the above-mentioned cytokines may play vital roles in HBV suppression, there may be multiple modes by which HBV is eliminated on TLR7 activation. Further studies are required to elucidate the exact pathway responsible in HBV clearance on triggering TLR7.

Eukaryotic genome is packaged into chromatin, which is subjected to dynamic structural alteration [30]. Post-translational modification (PTM) of core histone tails regulates gene expression spatiotemporally [31]. Site specific histone modifications can be read by effector proteins which delineate a specific cellular fate as highlighted through histone code hypothesis [30]. H3K4Me3 and H3K9Me3 are the transcription activation and repression signatures respectively. H3K9Ac also positively regulates transcription. Thus the global alterations of these epigenetic signatures reflect a major variation in cellular gene expression profile. Dynamic chromatin architecture is perturbed due to viral infection and an alteration of host posttranslational modification; fewer studies have embarked onto changes in host genome. Remarkably, Telbivudine, an anti HBV drug dramatically restores histone H3K4Me3 and H3K27Me3 of HepG2.2.15 cells akin to HepG2 cells [32]. Detailed investigation of host epigenetic changes as a sequel to TLR7 activation showed global repression of H3K9Me3 levels (Fig. 5a). This indicates a transcription de-repression scenario. Indeed we have seen upregulation of JNK responsive transcripts upon R837 treatment (Fig. 6a). This effect is however gene specific in broad perspective. Treatment of R837 to HepG2.2.15 cells reversed the epigenetic markers as well as transcription factor levels similar to HepG2 cells implicating that TLR7 activation possibly leads to viral reduction through suppression of HBV replication. These results further raise a possibility that antiviral response can be manifested by targeting the histone PTMs gene specifically and hence can be used as an epigenetic therapy.

Previous studies show Hepatitis B undergoes slow cell proliferation and induces a G1/S cell cycle arrest in HepG2.2.15 cells [16]. It is worth mentioning that HBx performs a dual activity of anti-proliferation and transactivation in HCC tissues. The study further showed that the effect of HBx on cell viability probably depends on the balance between pro-apoptotic and anti-apoptotic effects of NF-КB on different cell types [33]. Thus, the wild type HBx is responsible for the late G1 arrest, and upon TLR7 activation this arrest is partially released. The viral infection also triggers an upregulated expression of p53, which successfully retards the transition from G1 to S phase and prevents cell growth. The elevated p53 expression in liver biopsy samples of CHB patients re-affirmed the G1/S arrest induced by the virus. Previous studies have shown that serum p53 levels are higher in HBV- related cirrhotic patients compared to chronic HBV ones [34]. Interestingly, apart from alteration of p53 levels, Cyclin D1 and Cyclin E levels were dramatically modulated upon TLR7 activation implicating a role of p53 independent pathway regulating cell cycle. Thus, R837 treatment partially restores the cell cycle stages by removing the G1/S arrest and thus the antiviral response could be reestablished. No significant cell death was observed in the MTT assay. There was a clear indication of cell proliferation and there was increase in cell number at different time points on R837 treatment. The study on role of TLR7 in HBV infection has been shown in HepG2.2.15 cell line, harboring multiple copies of HBV genome, since the supernatant from the cell culture can infect chimpanzees intravenously, showing typical symptoms of human hepatitis, highlighting the efficiency of the model. Further, HepG2.2.15 cells have been used to study the pathogenesis of HBV and in elucidating the role of anti-viral drugs in HBV-related disorders [35–39]. Nevertheless, TLR7 levels and its role in HBV pathogenesis should be looked into other relevant systems like PHH cultures or NTCP-driven in vitro infection of different cell lines.

## Conclusion

The study thus shows that TLR7 plays a anti-viral role in HBV infection and holds a promising immuno-modulatory therapeutic value against this hepato-tropical viral disease.

## Additional file

Additional file 1: Figure S1a. AmRNA expression of different antiviral cytokines (closely associated with HBV clearance) that are upregulated on inciting TLR7. **Figure S1b.** Assessing the cytokines involved in HBV clearance. mRNA expression of different cytokines on addition of different protein blockers. Expression of IFN-β, TNF-α, IL-1β, IL-6 and IL-10 cytokines are curbed on blocking JNK pathway with its specific blocker SP600125.

## Abbreviations

CHB: Chronic HBV infection
ERK: Extracellular-signal-regulated kinases
HBV: Hepatitis B virus
HCC: Hepatocellular carcinoma
HCV: Hepatitis C virus
HIV: Human immunodeficiency virus
IFN: Interferon
IL-6: Interleukin-6
IRAKs: Interleukin (IL)-1-R–associated kinases
JNK: Jun n-terminal kinase
MAPK: Mitogen activating protein kinase
MyD88: Myeloid differentiation primary-response protein 88
NF- КB: Nuclear factor kappa-light-chain-enhancer of activated B cells
NLR: Nucleotide oligomerization domain (NOD)-like receptor
PI3K/AKT: Phosphatidylinositol-3 kinase/protein kinase B
PRR: Pattern recognition receptor
RLR: RIG-I-like receptor
SCID: Severe combined immunodeficiency disease
TLR: Toll like receptor
TNF: Tumor necrosis Factor
TRAF6: Tumor necrosis factor receptor (TNFR)-associated factor 6
WHV: Woodchuck hepatitis virus
