# Supplementary figures and images for: Anti-hepatitis B virus (HBV) response of imiquimod based toll like receptor 7 ligand in hbv-positive human hepatocelluar carcinoma cell line
Source: BMC Infect Dis. 2017 Jan 14;17:76. doi: 10.1186/s12879-017-2189-z (PMC5237519; doi:10.1186/s12879-017-2189-z)

## Slide 1
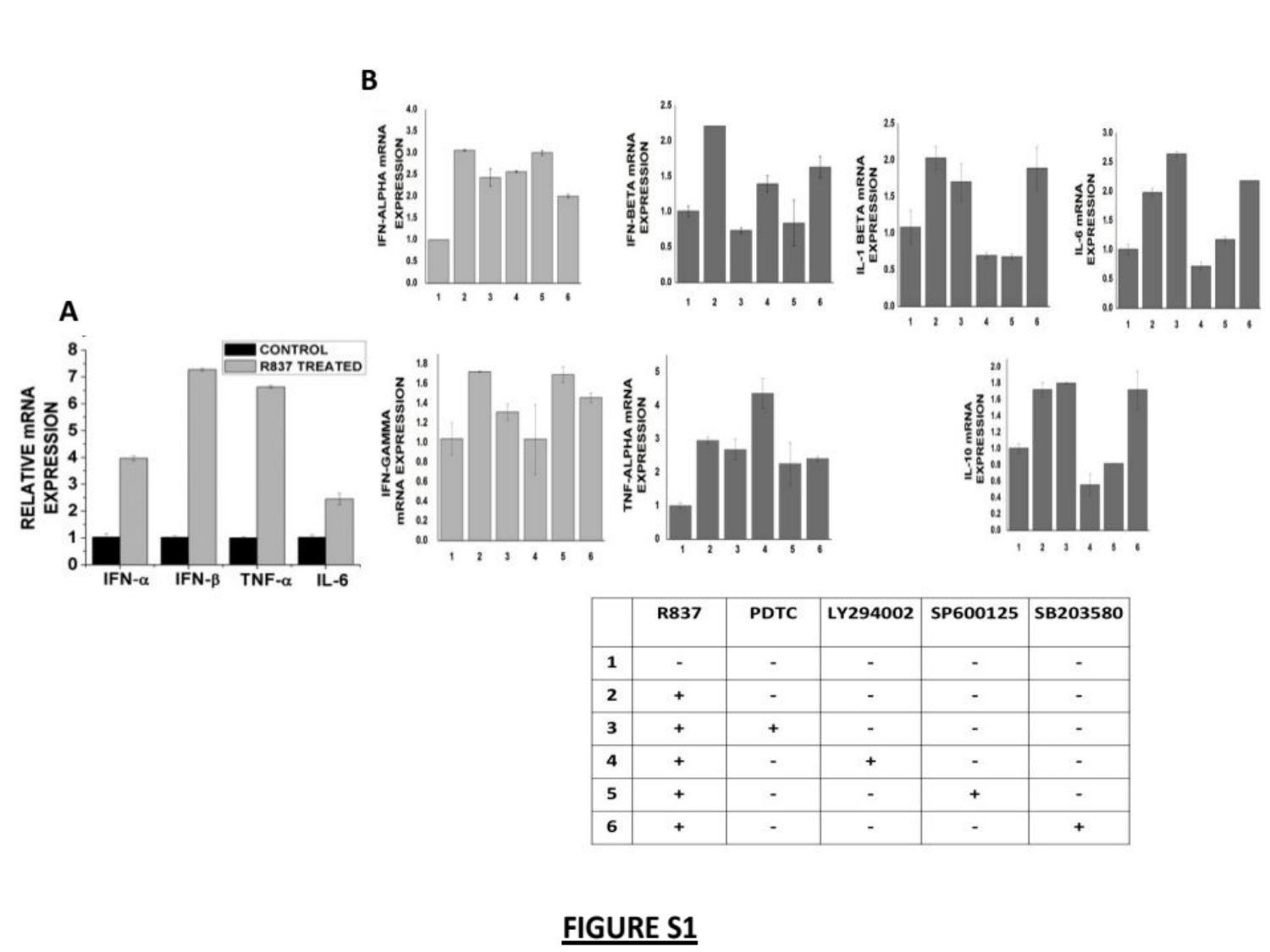

Supplement: Additional file 1: Figure S1a. — AmRNA expression of different antiviral cytokines (closely associated with HBV clearance) that are upregulated on inciting TLR7. Figure S1b. Assessing the cytokines involved in HBV clearance. mRNA expression of different cytokines on addition of different protein blockers. Expression of IFN-β, TNF-α, IL-1β, IL-6 and IL-10 cytokines are curbed on blocking JNK pathway with its specific blocker SP600125. [file 12879_2017_2189_MOESM1_ESM.pptx]
